# Psychiatric intervention on patients recovering from hospitalization due to Covid-19

**DOI:** 10.1192/j.eurpsy.2022.1308

**Published:** 2022-09-01

**Authors:** D. Machado, A. Borges, C. Laureano

**Affiliations:** Leiria Health Center, Psychiatry And Mental Health, Leiria, Portugal

**Keywords:** Covid-19, post-Covid psychiatric comorbidities, depression, anxiety, stress, mental health

## Abstract

**Introduction:**

According to recent reports Covid-19 patients may exhibit psychiatric co-morbidities that cause dysfunction, loss of autonomy and emotional suffering even after the physical illness is treated. Considering the high impact Covid-19 may have on mental health, we have created a psychiatric consultation dedicated to the study, observation and support of patients that developed mental illness after being hospitalized due to Covid-19.

**Objectives:**

We aim at 1) describe the profile of patients that developed psychiatric comorbidities following a hospitalization due to Covid-19 and 2) recognize and treat early psychiatric symptoms in Covid-19 patients.

**Methods:**

Based on what was described in other epidemic crisis, we established a semi-structured interview to evaluate several dimensions of the patients’ life that may have been affected by Covid-19 and that may impact on mental health. The interview included the *Depression, Anxiety and Stress Scale* (DASS-21), the *Impact of Event Scale – Revised* (IES-R) and the *Insomnia Severity Index* (ISI). Each patient was observed multiple times over several months. Our evaluation was done in parallel with consultations in Internal Medicine.

**Results:**

Most patients complained of symptoms directly related with the infection of SARS-CoV-2, namely fatigue, short breath and reduced tolerance to efforts. Importantly, many patients also reported *de novo* or aggravation of anxiety, stress, depression, sleep disturbances and grief often associated with feelings of existential emptiness and lack of purpose.

**Conclusions:**

Hospitalization due to Covid-19 has a high impact on mental health, raising important questions on purpose and emptiness. An early psychiatric intervention is highly recommended.

**Disclosure:**

No significant relationships.

